# Mutant Ubiquitin UBB+1 Induces Mitochondrial Fusion by Destabilizing Mitochondrial Fission-Specific Proteins and Confers Resistance to Oxidative Stress-Induced Cell Death in Astrocytic Cells

**DOI:** 10.1371/journal.pone.0099937

**Published:** 2014-06-18

**Authors:** Nambin Yim, Seung-Wook Ryu, Eun Chun Han, Jonghee Yoon, Kyungsun Choi, Chulhee Choi

**Affiliations:** 1 Cell Signaling and BioImaging Laboratory, Department of Bio and Brain Engineering, KAIST, Daejeon, Korea; 2 Graduate School of Medical Science and Engineering, KAIST, Daejeon, Korea; 3 KI for the BioCentury, KAIST, Daejeon, Korea; Mayo Clinic, United States of America

## Abstract

Mutant ubiquitin UBB+1 is observed in a variety of aging-related neurodegenerative diseases and acts as a potent inhibitor of the ubiquitin proteasome system (UPS). In the present study, we investigated the relationship between impaired UPS (using ectopic expression of UBB+1) and mitochondrial dynamics in astrocytes, which are the most abundant glial cells in the central nervous system. Immunocytochemistry and fluorescence recovery after photobleaching revealed that ectopic expression of UBB+1 induced mitochondrial elongation. We further demonstrated that overexpression of UBB+1 destabilized mitochondrial fission-specific proteins including Drp1, Fis1, and OPA3, but not the mitochondrial fusion-specific proteins Mfn1, Mfn2, and OPA1. The reduction in mitochondrial fission-specific proteins by UBB+1 was prevented by inhibiting the 26 S proteasome using chemical inhibitors, including MG132, lactacystin and epoxomicin. We then assessed the involvement of proteases that target mitochondrial proteins by using various protease inhibitors. Finally, we confirmed that either overexpression of UBB+1 or inhibiting the proteasome can protect astrocytic cells from H_2_O_2_-induced cell death compared with control cells. Our results suggest that UBB+1 destabilizes mitochondrial fission-specific proteins, leading to mitochondrial fusion and the subsequent resistance to oxidative stress. We therefore propose a protective role of UBB+1 overexpression or the proteasome inhibition in astrocytes in degenerative brains.

## Introduction

Impaired mitochondrial function has been implicated in the pathogenesis of various neurodegenerative maladies, including Alzheimer’s disease (AD) and Parkinson’s disease (PD) [1]. Mitochondria also play a significant role in the reciprocal signaling between astrocytes and neurons [2], and the dysfunction of astrocyte mitochondria can increase neuronal cell death [3], [4].

Mitochondria, critical regulators of various cellular activities, form a complex organized network controlled dynamically by the processes of fission and fusion [5]. Mitochondrial fission is mediated predominantly by a dynamin-related GTPase, Drp1 [6], [7] and its receptors, including Fis1, Mff, Mid49, and Mid51 [8], [9], [10]. OPA3, a mitochondrial outer membrane protein, can also mediate Drp1-independent mitochondrial fission [11]. Mitochondrial fusion is mediated by three specific proteins: Mfn1, Mfn2, and OPA1 [12], [13]. The alteration of mitochondrial dynamics is also commonly observed in a variety of neurodegenerative diseases and polyglutamine disorders [14]. Since mitochondrial proteins are subject to ubiquitin proteasome system (UPS)-mediated proteolysis, the activity of UPS may be tightly linked to mitochondrial dynamics and function.

Mutant ubiquitin B+1 (UBB+1) has been shown to suppress the function of UPS [15] and is one of the hallmarks of neurodegenerative diseases including AD, PD, Pick’s disease, and Huntington’s disease [16], [17], [18]. The ectopic expression of UBB+1 causes neuritic beading of the mitochondria and impairment of mitochondrial transport in cultured primary neurons [19], suggesting that potential cross talk occurs between UPS and mitochondria. Although dysregulation of UPS alters respiratory function and changes the expression of mitochondrial proteins [20], the role of UPS and ectopic UBB+1 expression in the maintenance of mitochondrial dynamics and function remains unclear.

Although UBB+1 exerts many detrimental and pathogenic effects, paradoxical roles of UBB+1 have also been reported, depending on the cell type and biological context [21]. For example, ectopic expression of UBB+1 did not induce neuronal loss in a transgenic animal model [22]. In addition, expression of UBB+1 acutely enhanced mitochondrial respiratory function, but effects were lost with long-term treatment [23]. The different biological effects of UBB+1 may be due to varying roles of UPS in different cell types, such as in neuronal and non-neuronal glial cells. To understand the complexity of the biological roles of UPS in the CNS, we investigated the effects of UBB+1 overexpression or UPS inhibition, focusing on mitochondrial dynamics in astrocytic cells. We demonstrated that UBB+1 protects astrocytic cells from oxidative stresses by regulating of mitochondrial dynamics, suggesting that the biological roles of UBB+1 in neurodegenerative diseases might be more complex than thought previously.

## Materials and Methods

### 1. Cell Culture

CRT-MG human astrocytoma cells were stably transfected with pEGFP and pEGFP-UBB+1 constructs, as previously described [21], [24], and cells were maintained in RPMI-1640 medium (Welgene, Seoul, Korea). The expression of UBB+1 was confirmed by immunoblotting using specific antibodies (Ab Frontier, Seoul, Korea). Primary human astrocytes were purchased from Lonza Clonetics (Walkersville, MD, USA) and expanded in accordance with the manufacturer’s instructions.

### 2. Reagents

The proteasome inhibitors MG132, lactacystin and epoxomicin were purchased from Calbiochem (La Jolla, CA, USA). Antibodies were purchased as follows: Drp1, OPA1, and Tom20 were obtained from BD Biosciences (San Diego, CA, USA), Fis1 from BioVision (Palo Alto, CA, USA), Mfn1 and GAPDH from Santa Cruz Biotechnology (Santa Cruz, CA, USA), Mfn2 from Sigma-Aldrich, and GFP from Cell Signaling Technology (Beverly, MA, USA). The OPA3 antibody was raised against a GST-fused partial protein [11]. Stabilized 10% hydrogen peroxide (H_2_O_2_) was purchased from Biopure (Ontario, Canada). Tetramethylrhodamine ethyl ester (TMRE) and Alexa Fluor 594-conjugated goat anti-mouse antibody were obtained from Molecular Probes (Eugene, OR, USA). Horseradish peroxidase (HRP)-conjugated secondary antibodies were purchased from Santa Cruz Biotechnology.

### 3. Analysis of Gene Expression

Total RNA was isolated from CRT-MG cells stably expressing pEGFP or pEGFP-UBB+1 using an RNeasy Mini Kit (Qiagen, Valencia, CA, USA). Complementary DNA (cDNA) was synthesized from 1 µg of total RNA using a high capacity RNA-to-cDNA kit (Applied Biosystems, Foster City, CA, USA). Quantitative real-time polymerase chain reaction (PCR) was performed in duplicate to determine the mRNA levels of Drp1, Fis1, OPA3, Mfn1, Mfn2, and OPA1 using the StepOne Real-Time PCR system (Applied Biosystems). Target mRNA levels were normalized to an endogenous reference gene (GAPDH). Data were quantified by the comparative threshold cycle method for calculating relative gene expression.

### 4. Immunoblotting

Cells were harvested, washed in ice-cold phosphate-buffered saline (PBS), and resuspended in adjusted volumes of buffer, provided with the M-Per mammalian protein extraction kit (Pierce, Rockford, IL, USA). The cells were incubated on ice for 30 min, and the lysates were then cleared by centrifugation (10,000×*g*, 10 min). The lysates were separated by 12% sodium dodecyl sulfate–polyacrylamide gel electrophoresis (SDS-PAGE), transferred to nitrocellulose membranes (Invitrogen, Carlsbad, CA, USA), and incubated with the appropriate antibodies. Blots were developed using an enhanced chemiluminescence system (Ab Frontier).

### 5. Measurement of Mitochondrial Membrane Potential (Δψm)

To assess mitochondrial membrane potential (*Δψm*), cells were stained with 2 µmol/L TMRE for 30 min at room temperature, washed with PBS, trypsinized, and resuspended in 200 µL binding buffer. Ten thousand cells were then analyzed with FACSCalibur (BD Biosciences) within 30 min of staining. Data were analyzed using the FlowJo software (Treestar, Inc., San Carlos, CA, USA).

### 6. RNA Interference and Gene Transfection

Small interfering RNA (siRNA) duplexes targeting Mfn1, Drp1, and a negative control random sequence siRNA were designed and synthesized by Bioneer (Daejeon, Korea). Target sequences were as follows: human Mfn1 5′-GUGUAGAUUCUGGUAAUGA-3′, human Drp1 5′-GAGGUUAUUGAACGACUCA-3′; negative control 5′-CCUACGCCAAUUUCGU-3′. The siRNAs were transiently transfected using Lipofectamine 2000 (Invitrogen) following the manufacturer’s instructions. One day after transfection, media were changed and cells were grown for an additional 48 h before experiments commenced. Transfection efficiency was confirmed by immunoblotting. Primary human astrocytes were transfected with pEGFP, pEGFP-UBB+1, or mito-DsRed using Effectene Transfection Reagent (Qiagen) according to manufacturer’s instructions.

### 7. Immunocytochemistry and Confocal Microscopic Analysis

Cells grown in 2-well chamber slides were fixed with 4% paraformaldehyde for 15 min, permeabilized with 0.15% Triton X-100 in PBS for 15 min, and then blocked with 3% bovine serum albumin in PBS for 45 min. The slides were incubated with Tom20 primary antibodies, followed by goat anti-mouse IgG antibody conjugated to Alexa Fluor 594. Images were captured using a Zeiss LSM 510 confocal microscope with 40× Apochromat objectives (Carl Zeiss, Thornwood, NY, USA). All incubations were carried out at room temperature.

### 8. Measurement of Mitochondrial Network Connectivity

The mitochondrial complex network was evaluated using fluorescence recovery after photobleaching (FRAP), which provides a quantitative measure of the volume of single units of the mitochondrial networks. Briefly, live cells were imaged using an LSM510 imaging station (Carl Zeiss). Small regions of interest (ROIs) of identical size containing Mito-DsRed-expressing cells were photobleached using a 30 mV argon laser set to 594 nm (with a 20% laser power output and 100% transmission) until the fluorescence intensity of the region reached the basal level. The region was then monitored for the recovery of DsRed fluorescence. The fluorescence intensity was normalized to the ROI in the first image of the series, and the fluorescence intensity recovery rates were plotted.

### 9. Detection of Cell Death

Cells were seeded in 12-well plates and washed twice with serum free RPMI-1640 medium. After treatment with H_2_O_2_, the supernatants were analyzed with a lactate dehydrogenase release assay (Promega, Madison, WI, USA) according to the manufacturer’s protocol.

### 10. Statistical Analysis

Data are presented as the mean ± standard deviation (SD). The significance of the difference between two independent samples was determined by Student’s *t*-test. Groups were compared using one-way analysis of variance (ANOVA), with Tukey’s post hoc test applied to the significant main effect.

## Results

### 1. Ectopic Expression of UBB+1 Induces Mitochondrial Elongation by Reducing the Expression of Fission-specific Proteins

**Figure 1:**
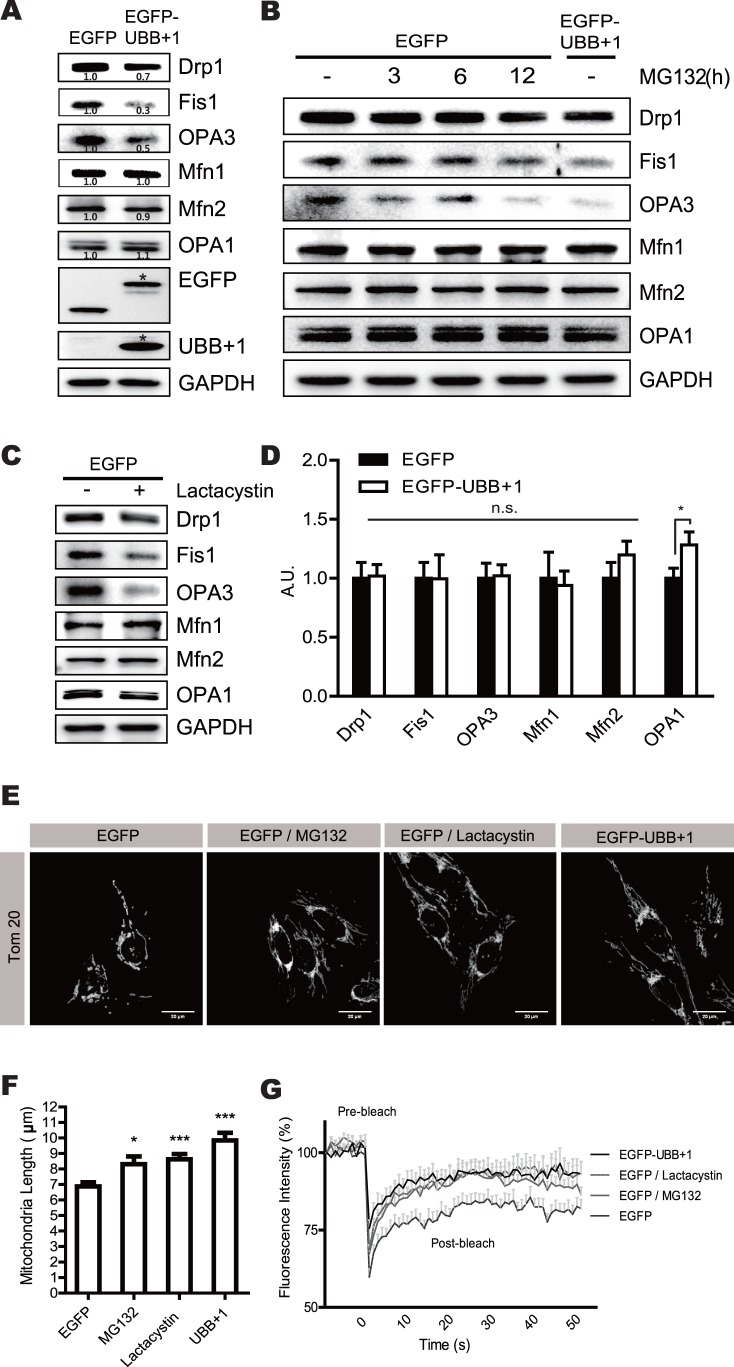
The regulation of mitochondrial dynamics by proteasome inhibition and ectopic expression of UBB+1 in CRT-MG human astrocytes. *A*, CRT-MG cells stably transfected with pEGFP or pEGFP-UBB+1 were harvested. Western blotting was carried out with antibodies against mitochondrial fission-specific (Drp1, Fis1, and OPA3) and fusion-specific proteins (Mfn1, Mfn2, and OPA1). GAPDH was used as a loading control, and all blots are representative of three independent experiments. Below numbers indicates the result of densitometry quantification. *B*, *C*, Cells were incubated in the absence or presence of 1 µmol/L MG132, a reversible proteasome inhibitor for 0–12 h, or 1 µmol/L lactacystin, an irreversible proteasome inhibitor, for 12 h. Cell lysates were analyzed by Western blotting for mitochondrial proteins. *D*, mRNA levels of Drp1, Fis1, OPA3, Mfn1, Mfn2, and OPA1 were assessed by real-time PCR. Statistical analysis was performed using Tukey’s post hoc test. Asterisks indicate significant differences compared with the control; *p<0.05. *E*, Mitochondrial morphology was analyzed with Tom20 staining by confocal microscopy in CRT-MG cells stably expressing pEGFP or pEGFP-UBB+1 after treatment with MG132 or lactacystin for 12 h (scale bar = 20 µm). *F*, The average mitochondrial length in single cells was measured using ImageJ software. Data are presented as the mean ± SEM, *n* = 30 cells. Turkey’s post hoc test was applied to significant group effects identified using ANOVA (*p*<0.0001). Asterisks indicate a statistically significant difference compared with the control; *p<0.05, ***p<0.001. *G*, The fluorescence intensity recovery rates after photobleaching are plotted. Cells were transfected with Mito-DsRed and incubated in the presence of 0.5 µmol/L MG132 or lactacystin for 12 h. Data are plotted as the mean ± SEM (n = 15–20 cells for each group).

To assess the effects of UBB+1 on mitochondrial dynamics, we first investigated the expression of proteins related to mitochondrial fission or fusion (Fig. 1*A* and Figure S1). Immunoblotting results demonstrated that the expression of the fission-related proteins Drp1, Fis1, and OPA3 was significantly reduced in UBB+1 stably transfected cells compared with the control. In contrast, the expression of the mitochondrial fusion proteins Mfn1, Mfn2, and OPA1 were not affected by the overexpression of UBB+1. Since UBB+1 is a potent inhibitor of the 26S proteasome [15], [25], we next used the pharmacological inhibitors MG132, lactcystin and epoxomicin to determine whether the UBB+1-mediated reduction in fission protein expression was due to the inhibition of UPS. As expected, the levels of Drp1, Fis1, and OPA3 significantly decreased after treatment with the inhibitors (Fig. 1*B, C* *,* and Figure S2), while the levels of Mfn1, Mfn2, and OPA1 were unaffected. To verify whether the effects on fission-specific proteins occurred at the transcriptional or translational level, we measured mRNA levels of these proteins using quantitative reverse transcription-PCR, and observed that the levels of Drp1, Fis1, and OPA3 mRNA were comparable between the control and UBB+1 cells (Fig. 1*D*). These results clearly suggest that the reduction in the expression of mitochondrial fission-specific proteins by ectopic expression of UBB+1 occurs at the translational or posttranslational level in a proteasome-dependent manner.

We next examined the effects of UBB+1 on mitochondrial dynamics by analyzing mitochondrial morphology in the UBB+1 stable cells (Fig. 1*E*). The mitochondria in cells overexpressing UBB+1 were typically more elongated in shape compared with the control cells. Importantly, similar effects were observed in cells treated with MG132 or lactacystin. In addition, the average length of mitochondria in UBB+1 stable cells or those treated with proteasome inhibitors was significantly longer than those in control cells (Fig. 1*F*). Since mitochondria exhibited an elongated morphology, we next assessed mitochondrial fission activity using FRAP analysis. The photobleached fluorescence was recovered within 10 s in the control cells, while the recovery time in UBB+1 cells or those with proteasome inhibitors was significantly shorter (Fig. 1*G*).

**Figure 2:**
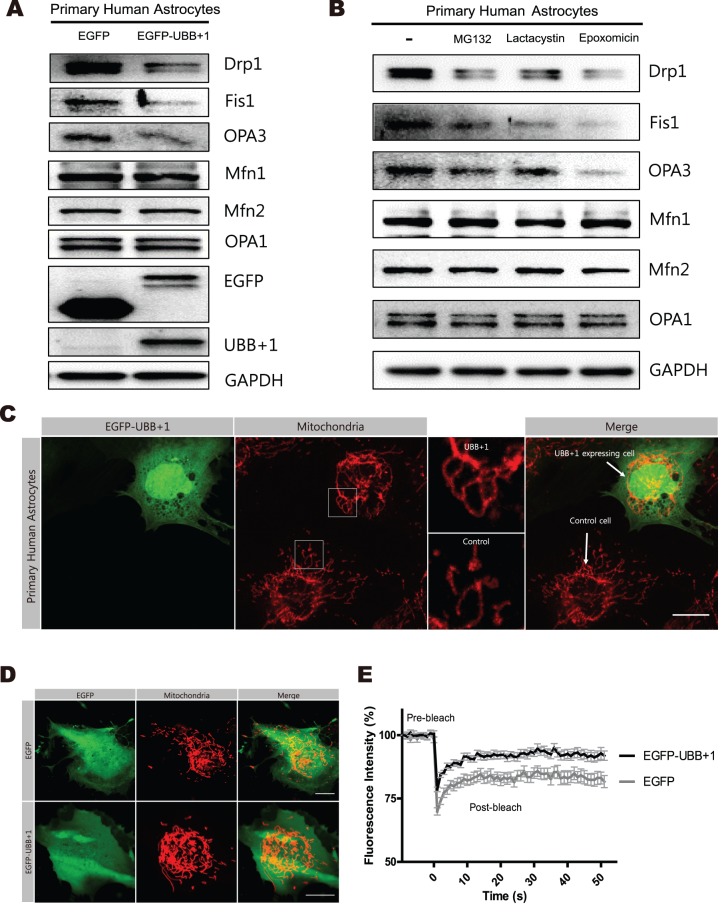
The regulation of mitochondrial dynamics by transient expression of UBB+1 and proteasome inhibition in primary human astrocytes. *A,* Astrocytes were transiently transfected with pEGFP or pEGFP-UBB+1. After 72 hrs, cells were lysed and analyzed by Western blotting for mitochondrial proteins (Drp1, Fis1, OPA3, Mfn1, Mfn2, and OPA1), EGFP, and UBB+1. GAPDH was used as a loading control, and all blots are representative of three independent experiments. *B,* Cells were incubated in the absence or presence of 1 µmol/L MG132, a reversible proteasome inhibitor for 12 h, 1 µmol/L lactacystin, an irreversible proteasome inhibitor, for 12 h, or 1 µmol/L epoxomicin, a highly specific proteasome inhibitor. Cell lysates were analyzed by Western blotting for mitochondrial proteins. *C.* Mitochondrial morphology was analyzed after staining for Tom20 protein by confocal microscopy in primary human astrocytes after 72 h transiently transfection with pEGFP-UBB+1. A higher magnification views of mitochondrial image in the white square are presented in the each right side of the images. (scale bar = 20 µm). *D, E,* Primary human astrocytes were co-transfected with pEGFP and mito-DsRed, or pEGFP-UBB+1 and mito-DsRed. After 72 hrs, confocal microscopy was used to analyze the mitochondrial morphology. And the fluorescence intensity recovery rates after photobleaching are plotted. Data are plotted as the mean ± SEM. (n = 15–20 cells for each group).

In addition, we investigated the effects of UBB+1 on mitochondrial dynamics in primary human astrocytes. Immunoblotting analysis revealed that the expression of Drp1, Fis1, and OPA3 was significantly reduced in the cells transiently transfected with UBB+1 construct or treated with various 26S proteasome inhibitors (Fig. 2*A, B*). The mitochondria in primary astrocytes transiently transfected with EGFP-UBB+1 were more elongated in shape compared with control cells (Fig. 2*C, D*). FRAP analysis confirmed increased mitochondrial fission activity in cells transfected with UBB+1 compared to normal control (Fig. 2*E*).

### 2. Ectopic Expression of UBB+1 Protects Astrocytic Cells from Oxidative Stress-induced Cell Death

**Figure 3:**
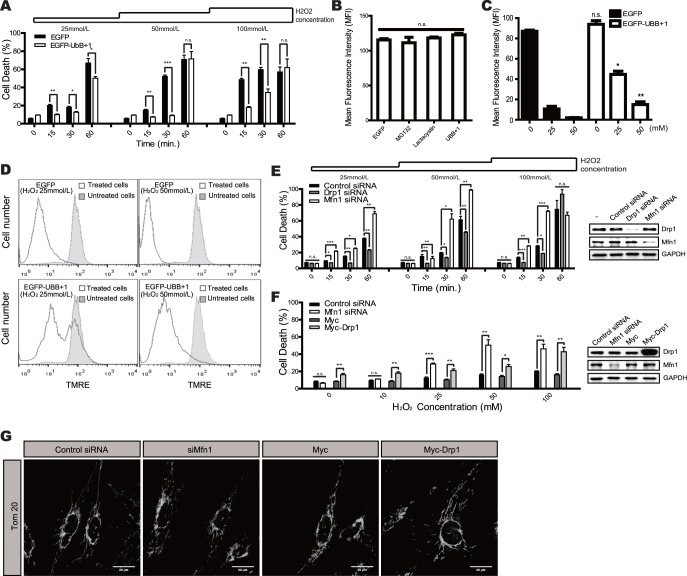
Effects of UBB+1 on oxidative stress-induced cell death. ***A***, CRT-MG cells stably expressing pEGFP and pEGFP-UBB+1 were incubated in the absence or presence of H_2_O_2_ (0–100 mmol/L) for different time periods as indicated (0–60 min). After stimulation, cell death was assessed using an LDH assay. Data are presented as the mean ± SEM (*n* = 3), and Turkey’s post hoc test was applied to significant group effects (*p*<0.0001) identified by ANOVA. Asterisks indicate a significant difference compared with the control: *p<0.05, **p<0.01, ***p<0.001. ***B***, Cells were stained with TMRE, and the mean fluorescence intensity was determined using FACS analysis. The graph represents the mitochondrial membrane potential Δψ. Data are presented as the mean ± SEM (*n* = 3), and Turkey’s post hoc test was applied to significant group effects identified by ANOVA. ***C***, The mean fluorescence intensity of TMRE staining was assessed after treatment with H_2_O_2_, and the graph represents the mitochondrial membrane potential Δψ. Data are presented as the mean ± SEM (*n* = 3), and Turkey’s post hoc test was applied to significant group effects identified by ANOVA. Asterisks indicate a significant difference between UBB+1-expressing and control cells. ***D***, CRT-MG cells stably expressing pEGFP or pEGFP-UBB+1 were treated with 25 or 50 mmol/L H_2_O_2_ for 15 min, and mitochondrial membrane potential was assessed using TMRE. Filled, untreated cells; lined, treated cells. ***E***, CRT-MG cells stably expressing EGFP were transfected with siRNA against Drp1, Mfn1, or the negative control, and stimulated with 0–100 mmol/L H_2_O_2_ for 0–60 min as indicated. Efficient knockdown of Drp1 and Mfn1 was confirmed by Western blotting. ***F***, CRT-MG cells stably expressing UBB+1 were transfected with Mfn1 siRNA, control siRNA, Drp1-overexpressing pCMV-Myc or pCMV-Myc. Cells were then stimulated with 0–100 mmol/L H_2_O_2_ for 30 min. Efficient knockdown of Mfn1 and overexpression of Drp1 were confirmed by Western blotting. After treatment, cell death was assessed by an LDH assay. Data are presented as the mean ± SEM (*n* = 3); Turkey’s post hoc test was applied to significant group effects (*p*<0.0001) identified by ANOVA. Asterisks indicate a significant difference compared with the control: *p<0.05, **p<0.01, ***p<0.001. ***G***, The mitochondrial morphology was analyzed after staining for Tom20 in UBB+1 expressing cells transfected with control siRNA, Mfn1 siRNA, Drp1-overexpressing pCMV-Myc or pCMV-Myc.

Mitochondrial dynamics are involved with the cellular susceptibility to death signals [26], [27]. We therefore hypothesized that ectopic expression of UBB+1 might affect cellular vulnerability to cell death by inducing mitochondrial elongation. We therefore assessed cell death in UBB+1 overexpressing and control cells after treatment with different doses of H_2_O_2_ for varying time periods (Fig. 3*A*). Incubation with H_2_O_2_ induced significant cell death in a dose- and time-dependent manner, while overexpression of UBB+1 significantly abrogated these effects. To confirm the protective effects of UBB+1, we next assessed changes in mitochondrial membrane potential using TMRE staining (Fig. 3*B*). TMRE fluorescence, an indicator of the mitochondrial membrane potential (*Δψm*), was not different between the different cell types, confirming that ectopic expression of UBB+1 did not induce cell death in astrocytic cells. The mean fluorescence intensity (MFI) of TMRE decreased upon treatment with H_2_O_2_ in a dose-dependent manner in the control cells. In contrast, the MFI in UBB+1 cells was significantly higher than in control cells (Fig. 3*C, D*).

We next examined whether the reduced cell death of UBB+1 stable cells was due to altered mitochondrial dynamics. We induced changes in mitochondrial dynamics toward fusion or fission using RNA interference against Drp1 and Mfn1, respectively. As expected, reducing Drp1 expression protected cells from H_2_O_2_-induced cell death, while reducing levels of Mfn1 increased oxidative stress-induced cell death (Fig. 3*E*). Next, we induced mitochondrial fission in UBB+1 stable cells by overexpressing Drp1 or silencing Mfn1, and assessed the sensitivity to H_2_O_2_-induced cell death (Fig. 3*F*). Under normal conditions, UBB+1 stable cells are relatively resistant to H_2_O_2_-induced cell death, even at high concentrations. However, the ectopic expression of Drp1 or the silencing of Mfn1 sensitized UBB+1 cells to oxidative stress. In addition, changes in the mitochondrial morphology of these cells were confirmed by confocal microscopy and FRAP analysis. (Fig. 3*G* and Figure S3). These results suggest that overexpression of UBB+1 may protect astrocytic cells from oxidative stress-induced cell death by regulating mitochondrial dynamics.

## Discussion

In the present study, we demonstrated that the overexpression of UBB+1 altered mitochondrial dynamics to fused and elongated states, and conferred cellular resistance to oxidative stress-induced cell death. In addition, the protective effects of UBB+1 overexpression or UPS blockade were due to the enhanced fusion activity of mitochondria. Although it is premature to conclude that UPS blockade or UBB+1 expression confers protective effects, our data clearly provide intriguing evidence that UBB+1 might have a protective role in aging or degenerating brains.

Dysfunction of UPS and impaired mitochondrial dynamics are important hallmarks of aging-related or disease-associated degeneration of neuronal cells. In addition, the impaired function of UPS is linked to mitochondrial dysfunction in neurons and is partially responsible for the increased susceptibility to cell death [14], [23], [28]. UBB+1 also plays a role in impaired mitochondrial function and enhanced cell death in neuroblastoma cell lines and cultured primary mouse neurons [19], [25]. UBB+1 also interacts with the ubiquitin-conjugating enzyme E2-25K/Hip-2 and plays a crucial role in amyloid-β neurotoxicity [29], [30]. In contrast, however, a recent study demonstrated that UBB+1 decreased the formation of amyloid plaques in a transgenic mouse model of AD [31]. Decreased activity of the UPS was hypothesized to delay the production of amyloid-β peptide by inhibiting β-secretase activity [32]. Nevertheless, our data demonstrate that the expression of UBB+1 induces mitochondrial elongation by inhibiting the 26S proteasome and destabilizing fission-specific proteins, and protects astrocytes from oxidative stress. Previous studies in a genetic model of UBB+1 showed no increased cell death in the neuronal cells [22], while another study reported central respiratory failure due to brain stem pathology, but only after long-term expression of UBB+1 [23]. These contradictory results from different models may be due to nonlinear dose-dependent effects of UBB+1. At low concentrations, UBB+1 acts preferentially as a substrate for ubiquitin fusion degradation, rather than as a proteasome inhibitor [15], [33], [34], and can be specifically degraded by ubiquitin C-terminal hydrolase L3 [35].

Under specific conditions, blocking UPS or overexpressing UBB+1 might exert different biological effects in astrocytes and neurons. Our data support the notion that ectopic expression of UBB+1 has no effect on cell viability or mitochondrial membrane potential in astrocytic cells. Furthermore, overexpression of UBB+1 or inhibition of UPS protected astrocytic cells from oxidative stress by regulating mitochondrial dynamics. Indeed, UBB+1 was induced in astrocytes by active caspase-3 in conditions of stress such as ischemia [36]. Although it is premature to conclude that UBB+1 has protective effect in astrocytes, these findings suggest that it may confer cellular protection by modulating mitochondrial dynamics in a cell-specific manner.

UBB+1 has been reported to increase the expression of the heat shock proteins Hsp40 and Hsp70 in human neuroblastoma cells in proteasome-dependent manner, which may protect the cells from oxidative stress [37]. Hsp40 and Hsp70 are key components in the import and folding of various mitochondrial proteins [38], [39], and they protect cells from premature cell death induced by various noxious stimuli [40], [41]. Since defects in Hsp70 can cause the aggregation of mitochondria in yeast [42], the destabilization of mitochondrial fission-specific proteins and increased expression of heat shock proteins induced by UBB+1 may work in concert to protect cells from oxidative stress. However, additional studies are needed to confirm this hypothesis.

Dysregulation of mitochondrial dynamics is frequently observed in the brains of animals and patients with degenerative diseases. Levels of Drp1 decrease significantly in animal models of AD and cause synaptic loss induced by amyloid-β-derived diffusible ligands [43]. Although Drp1-mediated disruption of mitochondrial fission inhibits cytochrome *c* release and prevents cell death [26], [44], [45], a sustained imbalance of mitochondrial dynamics is generally detrimental [14]. Modification of Drp1 has been reported to be involved in neuronal injury in brains of human Alzheimer’s disease patients [46]. However, reducing Drp1 stability by abnormal and continuous UBB+1 expression could ultimately cause pathological problems, such as the synaptic loss of neurons in neurodegenerative diseases.

In conclusion, the inhibition of UPS and overexpression of UBB+1 decreased the expression of the mitochondrial fission-specific proteins Drp1, Fis1, and OPA3, accompanied by increased mitochondrial fusion activity in human astrocytic cells, which conferred cellular resistance to oxidative stress-induced cell death. Based on these observations, we proposed that ectopic expression of UBB+1 might be essential for cellular resilience to oxidative stress by regulating mitochondrial dynamics.

## Supporting Information

Figure S1 The western blot analysis results for mitochondrial fission proteins in CRT-MG cells stably transfected with pEGFP or pEGFP-UBB+1 were quantified by densitometry. Data are presented as the mean ± SEM (*n* = 3).
(EPS)

Figure S2 The regulation of mitochondrial dynamics by various proteasome inhibitors. CRT-MG cells stably transfected with pEGFP were incubated in the absence or presence of lactacystin, epoxomicin, or MG132 (in final concentration of 1 µmol/L) for 12 h. Cell lysates were analyzed by western blotting for mitochondrial proteins.
(EPS)

Figure S3 CRT-MG cells stably expressing pEGFP-UBB+1 were co-transfected with mito-DsRed and Mfn1 siRNA or Drp1-overexpressing pCMV-Myc constructs. For control experiment, same cells were transfected with mito-DsRed and control siRNA or pCMV-Myc vectors. After 48 h, confocal microscopy was used to analyze the mitochondrial morphology. And the fluorescence intensity recovery rates after photobleaching are plotted. Data are plotted as the mean ± SEM (n = 20).
(EPS)

## References

[1] Schon EA, Przedborski S (2011) Mitochondria: the next (neurode) generation. Neuron 70: 1033–1053. 21689593 10.1016/j.neuron.2011.06.003 PMC3407575

[2] Calabrese V, Scapagnini G, Giuffrida Stella AM, Bates TE, Clark JB (2001) Mitochondrial involvement in brain function and dysfunction: relevance to aging, neurodegenerative disorders and longevity. Neurochem Res 26: 739–764. 11519733 10.1023/a:1010955807739

[3] Nedergaard M, Dirnagl U (2005) Role of glial cells in cerebral ischemia. Glia 50: 281–286. 15846807 10.1002/glia.20205

[4] Voloboueva LA, Suh SW, Swanson RA, Giffard RG (2007) Inhibition of mitochondrial function in astrocytes: implications for neuroprotection. J Neurochem 102: 1383–1394. 17488276 10.1111/j.1471-4159.2007.4634.x PMC3175820

[5] Chan DC (2006) Mitochondria: dynamic organelles in disease, aging, and development. Cell 125: 1241–1252. 16814712 10.1016/j.cell.2006.06.010

[6] Kageyama Y, Zhang Z, Sesaki H (2011) Mitochondrial division: molecular machinery and physiological functions. Curr Opin Cell Biol 23: 427–434. 21565481 10.1016/j.ceb.2011.04.009 PMC3379813

[7] Tamura Y, Itoh K, Sesaki H (2011) SnapShot: Mitochondrial dynamics. Cell 145: 1158, 1158 e1151. 10.1016/j.cell.2011.06.018 PMC3518382 21703455

[8] Yoon Y, Krueger EW, Oswald BJ, McNiven MA (2003) The mitochondrial protein hFis1 regulates mitochondrial fission in mammalian cells through an interaction with the dynamin-like protein DLP1. Mol Cell Biol 23: 5409–5420. 12861026 10.1128/MCB.23.15.5409-5420.2003 PMC165727

[9] Otera H, Wang C, Cleland MM, Setoguchi K, Yokota S, et al (2010) Mff is an essential factor for mitochondrial recruitment of Drp1 during mitochondrial fission in mammalian cells. J Cell Biol 191: 1141–1158. 21149567 10.1083/jcb.201007152 PMC3002033

[10] Palmer CS, Osellame LD, Laine D, Koutsopoulos OS, Frazier AE, et al (2011) MiD49 and MiD51, new components of the mitochondrial fission machinery. EMBO Rep 12: 565–573. 21508961 10.1038/embor.2011.54 PMC3128275

[11] Ryu SW, Jeong HJ, Choi M, Karbowski M, Choi C (2010) Optic atrophy 3 as a protein of the mitochondrial outer membrane induces mitochondrial fragmentation. Cell Mol Life Sci 67: 2839–2850. 20372962 10.1007/s00018-010-0365-z PMC11115811

[12] Chen H, Detmer SA, Ewald AJ, Griffin EE, Fraser SE, et al (2003) Mitofusins Mfn1 and Mfn2 coordinately regulate mitochondrial fusion and are essential for embryonic development. J Cell Biol 160: 189–200. 12527753 10.1083/jcb.200211046 PMC2172648

[13] Alexander C, Votruba M, Pesch UE, Thiselton DL, Mayer S, et al (2000) OPA1, encoding a dynamin-related GTPase, is mutated in autosomal dominant optic atrophy linked to chromosome 3q28. Nat Genet 26: 211–215. 11017080 10.1038/79944

[14] Itoh K, Nakamura K, Iijima M, Sesaki H (2013) Mitochondrial dynamics in neurodegeneration. Trends Cell Biol 23: 64–71. 23159640 10.1016/j.tcb.2012.10.006 PMC3558617

[15] Lindsten K, de Vrij FM, Verhoef LG, Fischer DF, van Leeuwen FW, et al (2002) Mutant ubiquitin found in neurodegenerative disorders is a ubiquitin fusion degradation substrate that blocks proteasomal degradation. J Cell Biol 157: 417–427. 11980917 10.1083/jcb.200111034 PMC2173284

[16] Fischer DF, De Vos RA, Van Dijk R, De Vrij FM, Proper EA, et al (2003) Disease-specific accumulation of mutant ubiquitin as a marker for proteasomal dysfunction in the brain. FASEB J 17: 2014–2024. 14597671 10.1096/fj.03-0205com

[17] de Pril R, Fischer DF, Maat-Schieman ML, Hobo B, de Vos RA, et al (2004) Accumulation of aberrant ubiquitin induces aggregate formation and cell death in polyglutamine diseases. Hum Mol Genet 13: 1803–1813. 15198995 10.1093/hmg/ddh188

[18] Hol EM, van Leeuwen FW, Fischer DF (2005) The proteasome in Alzheimer’s disease and Parkinson’s disease: lessons from ubiquitin B+1. Trends Mol Med 11: 488–495. 16213790 10.1016/j.molmed.2005.09.001

[19] Tan Z, Sun X, Hou FS, Oh HW, Hilgenberg LG, et al (2007) Mutant ubiquitin found in Alzheimer’s disease causes neuritic beading of mitochondria in association with neuronal degeneration. Cell Death Differ 14: 1721–1732. 17571083 10.1038/sj.cdd.4402180 PMC3258508

[20] Livnat-Levanon N, Glickman MH (2011) Ubiquitin-proteasome system and mitochondria - reciprocity. Biochim Biophys Acta 1809: 80–87. 20674813 10.1016/j.bbagrm.2010.07.005

[21] Choi K, Park J, Lee J, Han EC, Choi C (2013) Mutant Ubiquitin Attenuates Interleukin-1β- and Tumor Necrosis Factor-α-Induced Pro-Inflammatory Signaling in Human Astrocytic Cells. PLoS ONE 8: e67891. 23844119 10.1371/journal.pone.0067891 PMC3700915

[22] Fischer DF, van Dijk R, van Tijn P, Hobo B, Verhage MC, et al (2009) Long-term proteasome dysfunction in the mouse brain by expression of aberrant ubiquitin. Neurobiol Aging 30: 847–863. 18760506 10.1016/j.neurobiolaging.2008.06.009

[23] Irmler M, Gentier RJ, Dennissen FJ, Schulz H, Bolle I, et al (2012) Long-term proteasomal inhibition in transgenic mice by UBB(+1) expression results in dysfunction of central respiration control reminiscent of brainstem neuropathology in Alzheimer patients. Acta Neuropathol 124: 187–197. 22730000 10.1007/s00401-012-1003-7 PMC3400757

[24] Lee J, Choi K, Choi C (2010) Delineating role of ubiquitination on nuclear factor-kappa B pathway by a computational modeling approach. Biochem Biophys Res Commun 391: 33–37. 19895789 10.1016/j.bbrc.2009.10.155

[25] De Vrij FM, Sluijs JA, Gregori L, Fischer DF, Hermens WT, et al (2001) Mutant ubiquitin expressed in Alzheimer’s disease causes neuronal death. FASEB J 15: 2680–2688. 11726544 10.1096/fj.01-0438com

[26] Lee YJ, Jeong SY, Karbowski M, Smith CL, Youle RJ (2004) Roles of the mammalian mitochondrial fission and fusion mediators Fis1, Drp1, and Opa1 in apoptosis. Mol Biol Cell 15: 5001–5011. 15356267 10.1091/mbc.E04-04-0294 PMC524759

[27] Shutt TE, McBride HM (2013) Staying cool in difficult times: Mitochondrial dynamics, quality control and the stress response. Biochim Biophys Acta 1833: 417–424. 22683990 10.1016/j.bbamcr.2012.05.024

[28] van Leeuwen FW, Gerez L, Benne R, Hol EM (2002) +1 Proteins and aging. Int J Biochem Cell Biol 34: 1502–1505. 12200043 10.1016/s1357-2725(02)00043-2

[29] Song S, Kim SY, Hong YM, Jo DG, Lee JY, et al (2003) Essential role of E2-25K/Hip-2 in mediating amyloid-beta neurotoxicity. Mol Cell 12: 553–563. 14527403 10.1016/j.molcel.2003.08.005

[30] Ko S, Kang GB, Song SM, Lee JG, Shin DY, et al (2010) Structural basis of E2-25K/UBB+1 interaction leading to proteasome inhibition and neurotoxicity. J Biol Chem 285: 36070–36080. 20826778 10.1074/jbc.M110.145219 PMC2975229

[31] van Tijn P, Dennissen FJ, Gentier RJ, Hobo B, Hermes D, et al (2012) Mutant ubiquitin decreases amyloid beta plaque formation in a transgenic mouse model of Alzheimer’s disease. Neurochem Int 61: 739–748. 22797007 10.1016/j.neuint.2012.07.007

[32] Kienlen-Campard P, Feyt C, Huysseune S, de Diesbach P, N’Kuli F, et al (2006) Lactacystin decreases amyloid-beta peptide production by inhibiting beta-secretase activity. J Neurosci Res 84: 1311–1322. 16941495 10.1002/jnr.21025

[33] van Tijn P, de Vrij FM, Schuurman KG, Dantuma NP, Fischer DF, et al (2007) Dose-dependent inhibition of proteasome activity by a mutant ubiquitin associated with neurodegenerative disease. J Cell Sci 120: 1615–1623. 17405812 10.1242/jcs.03438

[34] van Tijn P, Verhage MC, Hobo B, van Leeuwen FW, Fischer DF (2010) Low levels of mutant ubiquitin are degraded by the proteasome in vivo. J Neurosci Res 88: 2325–2337. 20336771 10.1002/jnr.22396

[35] Dennissen FJ, Kholod N, Hermes DJ, Kemmerling N, Steinbusch HW, et al (2011) Mutant ubiquitin (UBB+1) associated with neurodegenerative disorders is hydrolyzed by ubiquitin C-terminal hydrolase L3 (UCH-L3). FEBS Lett 585: 2568–2574. 21762696 10.1016/j.febslet.2011.06.037

[36] Zhang Y, Xiong M, Yan RQ, Sun FY (2010) Mutant ubiquitin-mediated beta-secretase stability via activation of caspase-3 is related to beta-amyloid accumulation in ischemic striatum in rats. J Cereb Blood Flow Metab 30: 566–575. 19844237 10.1038/jcbfm.2009.228 PMC2949147

[37] Hope AD, de Silva R, Fischer DF, Hol EM, van Leeuwen FW, et al (2003) Alzheimer’s associated variant ubiquitin causes inhibition of the 26S proteasome and chaperone expression. J Neurochem 86: 394–404. 12871580 10.1046/j.1471-4159.2003.01844.x

[38] Azem A, Oppliger W, Lustig A, Jeno P, Feifel B, et al (1997) The mitochondrial hsp70 chaperone system. Effect of adenine nucleotides, peptide substrate, and mGrpE on the oligomeric state of mhsp70. J Biol Chem 272: 20901–20906. 9252417 10.1074/jbc.272.33.20901

[39] Fan CY, Lee S, Cyr DM (2003) Mechanisms for regulation of Hsp70 function by Hsp40. Cell Stress Chaperones 8: 309–316. 15115283 10.1379/1466-1268(2003)008<0309:mfrohf>2.0.co;2 PMC514902

[40] Hayashi M, Imanaka-Yoshida K, Yoshida T, Wood M, Fearns C, et al (2006) A crucial role of mitochondrial Hsp40 in preventing dilated cardiomyopathy. Nat Med 12: 128–132. 16327803 10.1038/nm1327

[41] Morano KA (2007) New tricks for an old dog: the evolving world of Hsp70. Ann N Y Acad Sci 1113: 1–14. 10.1196/annals.1391.018 17513460

[42] Kawai A, Nishikawa S, Hirata A, Endo T (2001) Loss of the mitochondrial Hsp70 functions causes aggregation of mitochondria in yeast cells. J Cell Sci 114: 3565–3574. 11682615 10.1242/jcs.114.19.3565

[43] Wang X, Su B, Lee HG, Li X, Perry G, et al (2009) Impaired balance of mitochondrial fission and fusion in Alzheimer’s disease. J Neurosci 29: 9090–9103. 19605646 10.1523/JNEUROSCI.1357-09.2009 PMC2735241

[44] Cassidy-Stone A, Chipuk JE, Ingerman E, Song C, Yoo C, et al (2008) Chemical inhibition of the mitochondrial division dynamin reveals its role in Bax/Bak-dependent mitochondrial outer membrane permeabilization. Dev Cell 14: 193–204. 18267088 10.1016/j.devcel.2007.11.019 PMC2267902

[45] Germain M, Mathai JP, McBride HM, Shore GC (2005) Endoplasmic reticulum BIK initiates DRP1-regulated remodelling of mitochondrial cristae during apoptosis. EMBO J 24: 1546–1556. 15791210 10.1038/sj.emboj.7600592 PMC1142564

[46] Cho DH, Nakamura T, Fang J, Cieplak P, Godzik A, et al (2009) S-nitrosylation of Drp1 mediates beta-amyloid-related mitochondrial fission and neuronal injury. Science 324: 102–105. 19342591 10.1126/science.1171091 PMC2823371

